# Perspectives of metal-organic framework nanosystem to overcome tumor drug resistance

**DOI:** 10.20517/cdr.2022.76

**Published:** 2022-10-18

**Authors:** Huafeng Wang, Shi Li, Yiting Yang, Lei Zhang, Yinghao Zhang, Tianxiang Wei

**Affiliations:** ^1^School of Environment, Nanjing Normal University, Nanjing 210023, Jiangsu, China.; ^2^School of Chemistry and Materials Science, Nanjing Normal University, Nanjing 210023, Jiangsu, China.

**Keywords:** Metal-organic framework, drug resistance, drug delivery, cancer therapy, nanosystem

## Abstract

Cancer is one of the most harmful diseases in the world, which causes huge numbers of deaths every year. Many drugs have been developed to treat tumors. However, drug resistance usually develops after a period of time, which greatly weakens the therapeutic effect. Tumor drug resistance is characterized by blocking the action of anticancer drugs, resisting apoptosis and DNA repair, and evading immune recognition. To tackle tumor drug resistance, many engineered drug delivery systems (DDS) have been developed. Metal-organic frameworks (MOFs) are one kind of emerging and promising nanocarriers for DDS with high surface area and abundant active sites that make the functionalization simpler and more efficient. These features enable MOFs to achieve advantages easily towards other materials. In this review, we highlight the main mechanisms of tumor drug resistance and the characteristics of MOFs. The applications and opportunities of MOF-based DDS to overcome tumor drug resistance are also discussed, shedding light on the future development of MOFs to address tumor drug resistance.

## INTRODUCTION

Cancer ranks as the second leading cause of death worldwide among various diseases. According to statistics and estimates by the World Health Organization (WHO), there are 19.3 million new cancer cases and nearly 10 million deaths worldwide in 2020^[1]^. The global cancer burden is expected to be 28.4 million cases in 2040, although cancer treatment technologies including surgery, chemotherapy^[2-5]^, radiation therapy^[6]^, chemodynamic therapy (CDT)^[7,8]^, gene therapy^[9-11]^, photothermal therapy (PTT)^[12]^, and immunotherapy^[13-15]^ have shown great progress in prolonging survival of cancer patients. However, the multidrug resistance (MDR) of anticancer drugs seriously affects the therapeutic effect, leading to tumor recurrence and metastasis, which has become one of the main obstacles in tumor treatment^[16,17]^.

With the rapid progress of nanotechnology, multifunctional nanoparticles (NPs) enable selective delivery of therapeutic and/or diagnostic drugs to target tumor sites, making precision therapy possible. To date, many organic/inorganic nanomaterials^[18-20]^, including polysaccharides^[21,22]^, proteins^[23,24]^, polymers^[25]^, metals and metal oxides^[26,27]^, mesoporous silica^[28]^, engineering macrophages^[29]^, nucleic acid nanodevices^[30-32]^, and metal-organic frameworks (MOFs)^[33,34]^, have been extensively designed to address physicochemical issues associated with free drugs, tumor drug resistance, and biological barriers during delivery. Among them, MOFs, as an emerging class of nanomaterials, have gained the broad interest of many researchers owing to their high specific surface area, tunable porous structure, satisfactory stability, and biocompatibility. In particular, MOFs have shown great potential and advantages as nanocarriers for drug delivery and protection, such as encapsulation of drugs, direct assembly of drug molecules as organic ligands^[35]^, post synthesis^[33]^, and surface modification^[36]^. Up to now, the MOF-based drug delivery system (DDS) has been employed to deliver drugs such as cisplatin^[37]^, doxorubicin (DOX)^[38-40]^, biomolecular agents^[2,41-43]^, and immunosuppressants^[44,45]^. Both single and multiple drug delivery using MOF-based DDS is available^[36,46]^, and combined therapeutic approaches with multiple mechanisms are also commonly performed^[47]^. In this regard, MOFs, as the carrier in the nanosystem, could protect the drug from degradation, controllably release the drug at the tumor site, inhibit the expression of drug-resistant proteins and genes, and modify the physiological state of the tumor microenvironment, which could enhance the therapeutic effect and mediate the immune behavior against the drug-resistant behavior of cancer cells^[44]^.

To achieve better outcomes in MOF-based nanotechnology for cancer therapy, it is an urgent need to gain a deeper understanding of the mechanisms of tumor resistance and evaluate the potential as well as the challenges of MOF-based DDS in tumor resistance.

## MECHANISMS OF TUMOR RESISTANCE

**Figure 1:**
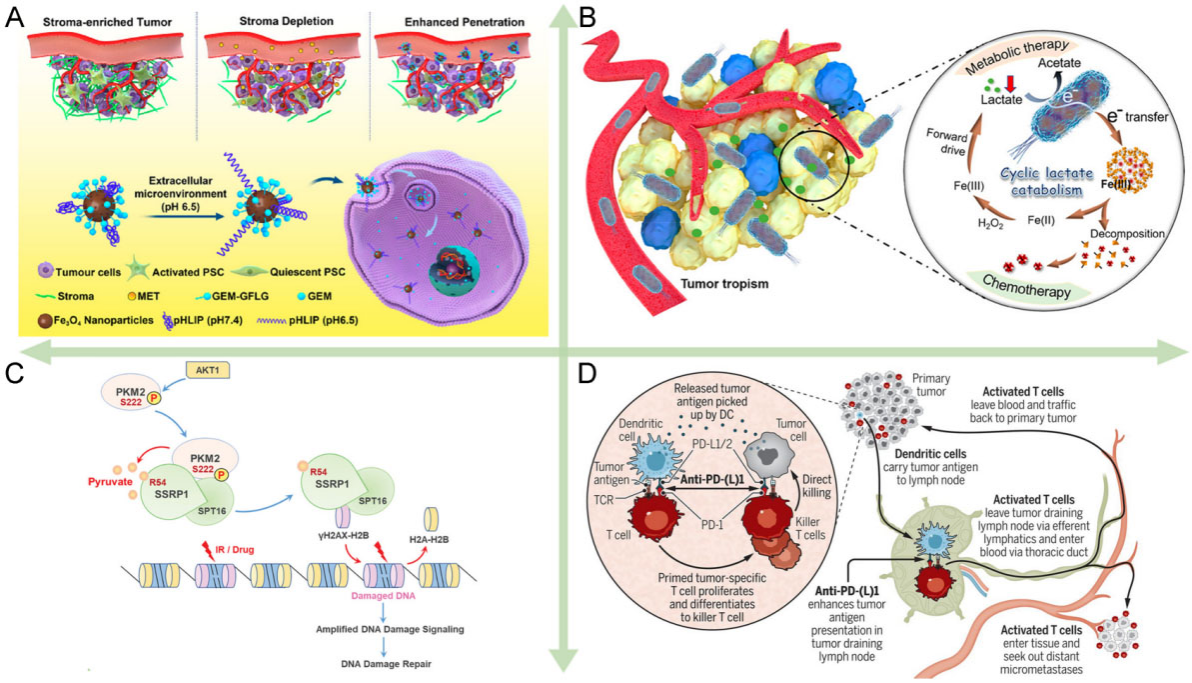
The major mechanisms of tumor resistance. (A) Biological barriers^[48]^. Reproduced with permission from ref^[48]^. Copyright 2020 American Chemical Society. (B) Drug inactivation^[40]^. Reproduced with permission from ref^[40]^. Copyright 2021 American Chemical Society. (C) DNA damage repair^[61]^. Reproduced with permission from ref^[61]^. Copyright 2022 the authors. (D) Immune evasion^[69]^. Reproduced with permission from ref^[69]^. Copyright 2020 Science.

MDR in tumors is complex and multifactorial, including biological barrier formed by the tumor microenvironment (TME)^[48-53]^ and overexpression of drug efflux transporter resulting in the inability to accumulate drugs intracellularly^[40,54-57]^, the drug inactivation due to the specific environment of gene control and metabolism, the resistance to apoptosis and deoxyribonucleic acid (DNA) damage repair^[58-63]^, and immune evasion^[64-71]^, as shown in Figure 1.

### Biological barriers

The biological barrier formed by the extracellular matrix (ECM) of the dense tumor site makes it difficult for drugs to reach the target site and is the main reason for human pancreatic ductal adenocarcinoma (PDAC) being one of the least curable and most malignant cancers. PDAC is characterized by the presence of a rich matrix. Pancreatic stellate cells secrete an excess of ECM proteins comprising the matrix with collagen, fibronectin, laminin, and glycoprotein as the main components^[51]^, following the activation by pro-fibrotic mediators including transforming growth factor *β* (TGF-*β*). The high density of matrix binds to a large number of stromal cells to form a biological barrier, and the two matrices work together to prevent drug penetration into the cancer cells. Recently, some researchers have developed new strategies to overcome the barrier. For example, Han *et al. *utilized metformin to disrupt the dense matrix by activating adenosine phosphate-activated kinase pathway, thereby promoting the penetration and therapeutic effect of the DDS [Figure 1A]^[48]^. Lv *et al.* showed that PDAC cells utilized gasdermin E to mediate resistance to digestive juices in the pancreatic microenvironment^[72]^.

Dense ECM is a distinctive feature of PDAC, but overexpression of drug efflux pumps is more prevalent in more cancer cells. The overexpression of drug efflux pumps is one of the essential mechanisms of MDR in cancer cells^[50,52]^, as various adenosine triphosphate (ATP)-binding cassette transporter family proteins are expressed on the cell membrane. Most of these proteins are ATP-driven multidrug efflux pumps such as MDR protein and P-glycoprotein (P-gp). After the intracellular drug binds to the transporter on the cell membrane, ATP hydrolysis drives a conformational change and pushes the drug out of the cell, resulting in the inability of the intracellular drug to accumulate in sufficient quantities to kill the cells^[73]^. Ruan *et al.* used nanocarriers to reverse drug resistance induced by overexpression of the drug efflux pumps by inhibiting mitochondrial ATP synthesis^[52]^. The cyclin-dependent kinase 6-phosphoinositide 3-kinase (CDK6-PI3K) signaling axis is an effective target for attenuating ATP binding cassette subfamily B member 1/P-gp-mediated MDR in cancer cells^[11,74,75]^.

### Drug inactivation

The cancer cells are continuously subjected to intense metabolic activity and exhibit hypoxia, high lactate content, and slightly acidic TME. Thereinto, lactate has been shown to be involved in downregulating the expression of the drug efflux pumps P-gp and reducing drug resistance^[40]^, while hypoxia causes the failure of many kinetic treatments that rely on oxygen for oxidative stress. In the drug-induced adverse factors, cancer cells will activate metabolic adaptations to eliminate the adverse factors and evade anticancer treatment [Figure 1B]. Activated metabolic adaptation consists of two major cellular pathways, mitochondrial oxidative phosphorylation (OXPHOS) to glycolytic ATP production and autophagy to recycle harmful substances within the cells^[54]^. To keep a higher level of metabolism, cancer cells maintain a stable antioxidant defense system internally with a relative balance between glutathione (GSH) and reactive oxygen species (ROS)^[55]^. Drugs entering the cells are often depleted by GSH and cannot increase ROS content to generate oxidative stress, which leads to the failure of dynamic therapy. Resistance based on GSH and ROS mechanisms has led to the inefficiency or even failure of most chemotherapeutic and kinetic treatments that rely on stimulating oxidative stress^[3,56,57,76]^.

Wang *et al.* demonstrated that nitric oxide (NO) could be used to reduce oxygen consumption by inhibiting cancer cell respiration, thereby eliminating hypoxia-induced chemoresistance of DOX^[56]^. Jiang *et al.* synthesized photosynthetic microcapsules to successfully complete sustained photosynthetic oxygenation in cancer cells, inducing lipid peroxidation and iron death processes to kill cancer cells^[76]^.

The emergence of secondary drug resistance has exacerbated the difficulty of treating cancer with some drugs. Secondary drug resistance indicates that, after being treated with one drug, cancer cells become resistant to another specific drug that was not previously used^[77]^; that is, its development is the dynamic clonal evolution of primary resistance to the drugs used. Aldonza *et al.* found that cancer cells with primary acquired resistance to the microtubule-stabilizing drug paclitaxel also tended to exhibit resistance to epidermal growth factor receptor-tyrosine kinase inhibitors (EGFR-TKIs), even though the cancer cells had never been exposed to this drug before^[77]^.

### Resistance to apoptosis and DNA damage repair

Cancer cells, which are generated from gene mutations in normal cells, can autonomously control the apoptotic program and enter an endless cell cycle. To induce DNA damage in cancer cells to cause cell necrosis and apoptosis is an overwhelming chemotherapeutic idea to destroy cancer cells. The chemotherapeutic drug cisplatin interacts with DNA to inhibit DNA replication and induce apoptosis killing cells. The chemotherapeutic drug DOX, a topoisomerase II (TOP2) inhibitor, inhibits DNA remodeling by inserting into double-stranded DNA and suppressing TOP2 activity. However, DNA damage repair (DDR) plays an important role in resistance to apoptosis mediated by anticancer drugs, including base excision repair, base mismatch repair, nucleotide excision repair, and DNA double-strand break repair^[78]^. Proactively enhanced DDR produces resistance of cancer cells to DNA damage-based therapies. Recently, Wu *et al.* observed that the products of glycolysis and exogenous pyruvate in cancer cells could enhance H2A variant H2AX serine (S) 139 (γH2AX) loading on chromatin, thereby promoting DNA repair [Figure 1C]^[61]^. Chen *et al.* employed a hypoxia-activated chemotherapeutic in cancer cells to downregulate the DNA repair protein, xeroderma pigmentosum group F, whose overexpression triggered resistance to cisplatin treatment^[3]^.

Cancer cells could prevent apoptosis through immune checkpoint blockade and autophagy^[58,60,79]^. Among them, immune checkpoint blockade tends to target extracellular immune cells for killing, while apoptosis is evaded intracellularly by autophagy and regulation of partial protein expression^[50]^. Cancer cells can suppress apoptosis by the overexpression of anti-apoptotic proteins (e.g., B-cell lymphoma-2 (BCL-2), BCL-XL, and myeloid cell leukemia-1 (MCL-1)) to downregulate or inactivate pro-apoptotic proteins (BCL-2-associated X, BCL-2-associated K, and BCL-2-related ovarian killer)^[59]^. Chen *et al.* used synthetic NPs to destabilize microtubules and prevent the formation of spindles in normal mitosis, leading to abnormal cell division and eventually apoptosis^[62]^. Wang *et al.* found that acetaldehyde dehydrogenase (ALDH2) gene was involved in mediating the RAF/MAPK signaling pathway, which is engaged in the regulation of apoptosis and drug sensitivity^[74]^.

### Immune evasion

Cancer immunotherapy has achieved clinical success and the use of T cells to kill cancer cells is a highly effective therapeutic mechanism. However, cunning cancer cells can evade the immune response by shaping immunosuppressive TME and immune checkpoint blockade. Joung *et al.* revealed that four genes, programmed death-ligand 1 (PD-L1), MCL-1, JunB proto-oncogene (*JUNB*), and β1,3-N-acetylglucosaminyltransferases (*B3GNT2*), affect the interaction of cancer cells with T cells, conferring resistance to T cell cytotoxicity^[64]^. Programmed cell death protein 1 (PD-1) on the T cells interacts with PD-L1 on the cancer cells to inhibit activation of T cells^[67]^. To address this problem, Topalian *et al.* proposed neoadjuvant PD-(L)1 blockade therapy to enhance systemic immunity against tumors and prevent recurrence [Figure 1D]^[69]^. In addition, Huang *et al.* summarized that multiple checkpoint blockades, including PD-1 and cytotoxic T lymphocyte antigen-4 (CTLA-4), developed an immunosuppressive TME in melanoma tumors^[65]^. Recently, Saha *et al.* revealed that cancer cells can interact with platelets entering TME to produce chemoresistance^[80]^. The platelet has been increasingly found to work as an important activator to induce epithelial-mesenchymal transition (EMT), while EMT is the primary and key event in promoting the distant spreading of metastatic tumor cells^[81]^. In another study, indoleamine 2,3-dioxygenase (IDO) in TEM was found to catalyze the metabolism of L-tryptophan to L-kynurenine, thus inhibiting the increase of effector T cells and promoting the growth of regulatory T (Treg) cells^[70]^.

## METAL-ORGANIC FRAMEWORK NANOSYSTEM

### Synthesis of MOFs and drug loading methods

**Figure 2:**
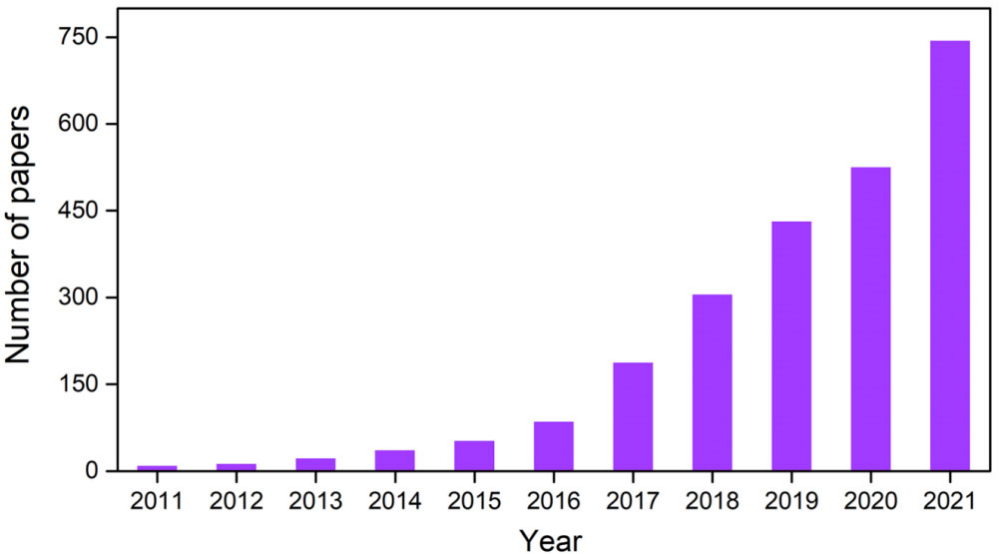
The number of publications in the last decade on the topic of therapy using MOFs according to Web of Science search.

MOFs are a class of crystalline porous materials with periodic network structures constructed by metal (cluster) nodes and organic ligands through coordination self-assembly^[82]^. Benefiting from the abundant species of metal (cluster) nodes and organic ligands, and the diverse coordination patterns between them, tens of thousands of MOFs have been reported. The research on MOFs in therapy has exhibited rapid growth in recent years [Figure 2].

Various synthetic methods provide flexibility in the synthesis of MOFs. Solvothermal and non-solvothermal methods are common approaches for the fabrication of MOFs. Solvothermal synthesis is typically performed at high temperatures or high pressure to dissolve the reagents and facilitate the synthesis. Non-solvothermal synthesis is performed at temperatures that are lower than the boiling point of the solvent and favorable for nucleation. Non-traditional synthetic approaches for drug delivery include microwave, sonication, mechanical milling, or electrochemical synthesis^[83]^.

**Figure 3:**
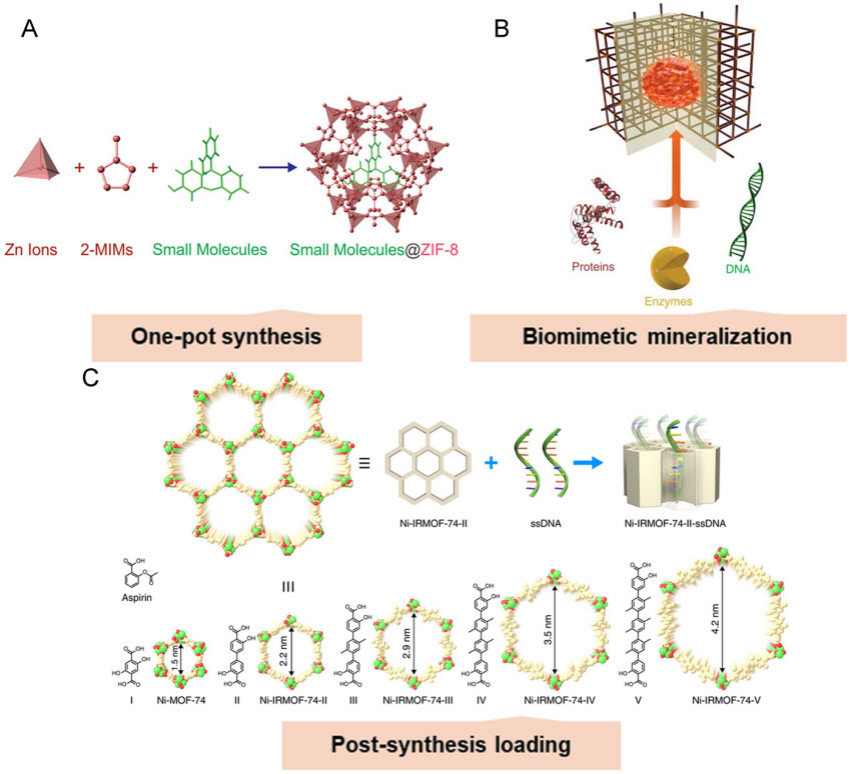
The common drug loading methods on the MOFs. (A) One-pot synthesis^[84]^. Reproduced with permission from ref^[84]^. Copyright 2014 American Chemical Society. (B) Biomimetic mineralization^[85]^. Reproduced with permission from ref^[85]^. Copyright 2015 the authors. (C) Post-synthetic loading^[86]^. Reproduced with permission from ref^[86]^. Copyright 2018 the authors.

The key challenge for the application of MOFs in the biomedical field lies not only in the precise control of their synthesis but also in the effect of surface affinity on their application behavior. Remarkably, the attractiveness of MOFs as drug delivery vehicles is mainly due to their special drug-carrying capacity. The drug loading efficiency is determined by the physical properties of the MOF (e.g., pore size, surface area, and spatial structures). The loading methods are essential to maximize loading and attain the ideal release. There are three common drug loading techniques for MOFs: one-pot synthesis^[84]^, biomimetic mineralization^[85]^, and post-synthesis loading^[86] ^[Figure 3].

One-pot synthesis is the co-precipitation of therapeutic molecules with MOFs during synthesis, resulting in a uniform distribution of drug agents in the pores of the MOF. One-pot synthesis is beneficial for the controlled release of drugs in the MOF, provided that the pore size of the MOF is small enough to limit the rapid diffusion of drugs through the MOF structure [Figure 3A]^[84]^. Biomimetic mineralization is valuable for loading biological agents such as protein and nucleic acid. Unlike one-pot synthesis, biomimetic mineralization utilizes bioagents as nucleation sites for MOF crystallization [Figure 3B]^[85]^. Specifically, the biomolecule partially interacts with the MOF building blocks, thereby promoting nucleation. Thus, the encapsulated biological molecules determine the sizes, morphologies, and crystallinities of the MOFs. Such an efficient encapsulation mechanism has been proved to protect bioagents from harsh chemical environments, heat, and degrading enzymes. Since the therapeutic drugs are integrated into the MOF, their release is dependent on the degradation of the MOF, which may lead to the sustained release and delayed activity of the encapsulated drugs. Post-synthesis loading involves the encapsulation of medicaments into the pores or the surface of the MOF after synthesis [Figure 3C]. This could be obtained commonly by mixing the MOF and medicaments in a solvent, followed by evaporation to remove the solvent^[86,87]^. Surface loading is typically dominated by unsaturated metal site interactions and electrostatic interactions and could also be implemented by attaching the drugs to the polymer-coated surface^[88]^. Unfortunately, surface loading normally leads to reduced drug loading in contrast to other approaches. Overall, the high porosity of the MOF provides space for the loading of guest molecules. The well-defined structure of MOFs has a clear relationship to their properties, which provides guidance for future modifications. Regardless of the encapsulation method employed, MOFs protect bioagents from degradation and expand potential avenues for clinical drug delivery.

### Advantages of MOFs

MOFs have not only excellent porous properties (e.g., high specific surface area and high porosity) but also many advantages such as structure design and function adjustment, so they have been widely applied in gas adsorption and separation^[89,90]^, chemical sensing^[91,92]^, heterogeneous phase catalysis, biological diagnosis^[93,94]^, and cancer therapy. In view of the flexibility in the design and synthesis of MOF materials, it is convenient to introduce primitives into MOFs through metal nodes, ligands, and guests to endow such materials with unique activities, thereby constructing multifunctional MOFs. Different from the simple loading of primitives by traditional heterogeneous porous materials, the structure of MOFs is ordered and observable, which is conducive to understanding the distribution of primitives in the framework and their interactions at the microscopic scale. Therefore, the structure-activity relationship between spatial structure and performance could be better understood. Furthermore, MOFs are tunable in structure and easily modified. It is easy to adjust the structure of MOFs and introduce various functional groups through crystal engineering and to exert a synergistic effect among the active centers through a specific spatial arrangement in MOFs, thereby regulating chemical interactions between multiple components within the framework. In addition, the rich and diverse pore structures of MOFs are not only conducive to material transport, but also can mimic the function of enzymes. Their unique cavities can generate a "spatial confinement effect" on the guest molecules, thereby influencing the behavior between the host and the guest from a kinetic aspect. Therefore, MOFs, as a class of excellent nanocarriers, are characterized by porosity, heterogeneous properties, and biocompatibility, which endow them with unique advantages in the fields of enzyme-like catalysis, disease diagnosis^[95]^, therapy^[96,97]^, and bioimaging.

### Potential biomedical application of MOFs

#### Drug delivery

**Figure 4:**
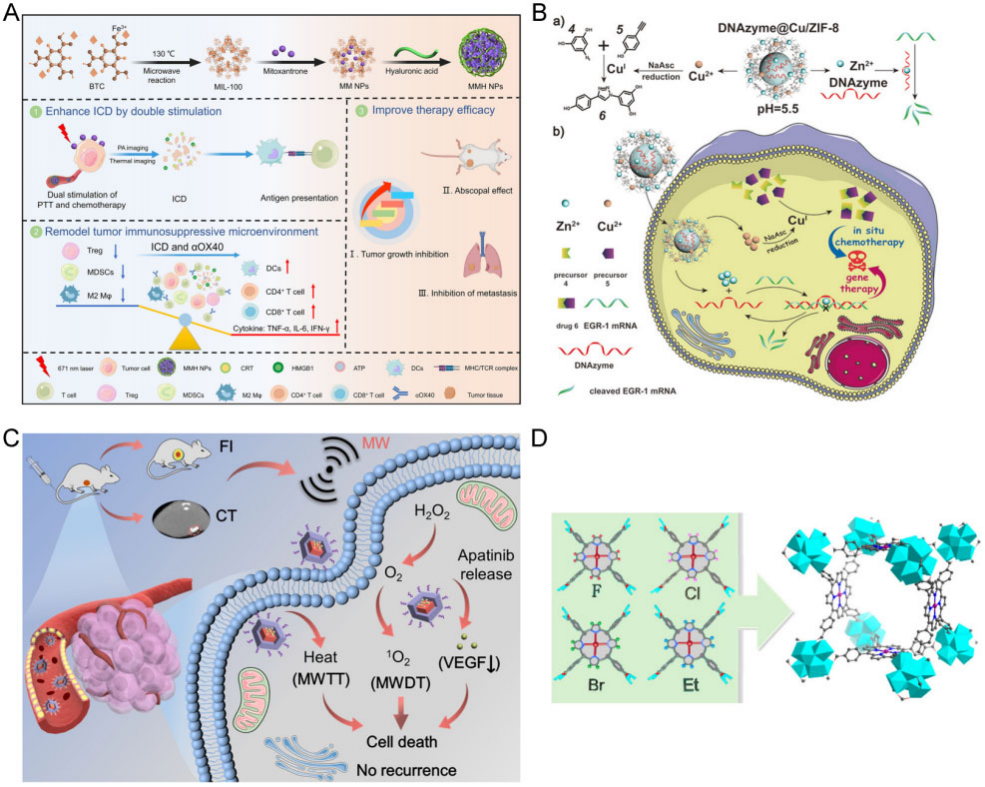
Summary of the major junctions of MOFs in biomedical application. (A) Drug delivery^[96]^. Reproduced with permission from ref^[96]^. Copyright 2021 American Chemical Society. (B) Intrinsic therapeutic MOF^[42]^. Reproduced with permission from ref^[42]^. Copyright 2021 Wiley-VCH. (C) Biological imaging^[102]^. MWTT, microwave thermal therapy; MWDT, microwave dynamic therapy; VEGF, vascular endothelial growth factor. Reproduced with permission from ref^[102]^. Copyright 2022 Elsevier. (D) Biomimetic catalysis^[105]^. Reproduced with permission from ref^[105]^. Copyright 2017 American Chemical Society.

To overcome the inherent limitations of therapeutic drugs and achieve targeted delivery and controllable release of drugs, the development of drug nanocarriers has become a research hotspot. One of the most essential considerations for drug nanocarriers is that the drugs must be released at a certain rate until the target site is reached, achieving the appropriate dose in a given time. The biological metal-organic framework (BioMOF), connecting MOF chemistry with bioscience, has become a drug delivery vehicle due to its high drug loading capacity and excellent biodegradability^[98,99]^. Recently, Ni *et al.* synthesized MIL-100 NPs loaded with chemotherapeutic drug mitoxantrone and hyaluronic acid (HA) [Figure 4A]^[93]^. The NPs targeted cancer cells with recognition of cluster of differentiation 44 (CD44) by HA, while co-injected anti-OX40 antibody (αOX40) reversed the immunosuppressive effect, allowing NPs to enter cancer cells favorably and release drug for chemotherapy. A bimetallic MOF for intracellular drug synthesis and self-sufficient therapy was designed [Figure 4B]^[42]^. Copper ions that were liberated from MOFs could catalyze the drug synthesis, killing tumors on site to minimize side effects on normal cells.

#### Biological imaging

The rapid development of bioimaging technology has contributed significantly to exploring the pathological characteristics and metabolic functions of biological tissues by providing vital equipment, which has greatly facilitated the diagnosis of diseases. More recently, MOF-based nanocomposites have been widely used in fluorescence imaging (FL), computed tomography (CT), and magnetic resonance imaging (MRI), as well as other fields, due to their simple functionalization, diverse structures and compositions, and large porosity^[100,101]^. Cheng *et al.* prepared a bimetallic Cu/Zn-MOF, further hollowed the multivalent (Cu^+/2+^/Mn^2+/4+^) structure after heating in manganese(II) acetylacetonate, and finally loaded indocyanine green (ICG). This MOF nanocarrier released Mn^2+^ for achieving MRI via a Fenton-like reaction and ICG for turn-on FL^[101]^. Li *et al.* synthesized a covalent organic framework cladding MOF (MOF@COF) encapsulated with Bi^3+^, Mn^2+^, and meso-Tetra (4-carboxyphenyl) porphyrin (TCPP) [Figure 4C]^[102]^. Upon reaching the tumor site, the nanocapsules degraded and Bi could be used for CT while TCPP was used for FL, ensuring extremely high imaging performance.

#### Biomimetic catalysis

An enzyme is a robust macromolecular biocatalyst that accelerates chemical reactions with unparalleled efficiency and specific selectivity under mild conditions. Thus, biomimetic catalysis for tumor therapeutic application is a highly attractive field with broad prospects for efficient catalysis^[103]^. The construction of functional systems that mimic natural enzymes is a remarkably popular goal. Especially, metalloporphyrins and their derivatives have been attracting attention as the catalytic centers of certain enzyme families. Feng *et al.* reviewed the precise synthesis of various MOFs using robust and multifunctional porphyrins as ligands and evaluated the efficient enzyme-like catalytic ability of porphyrin MOFs exhibited in oxidation catalysis, Lewis acid catalysis, electrocatalysis, and photocatalysis, providing ideas for the future synthesis of specific functional porphyrin MOFs and the development of their enzyme-like catalytic function^[104]^. Zr-MOF-based single substitution toward high-performance catalysis was proposed, achieving the molecular-level control of the chemical environment around the catalytic center [Figure 4D]^[105]^. Zhao *et al.* demonstrated the synthesis of Corrole-based MOF exhibits more efficient heterogeneous catalytic action than the porphyrin-based MOFs^[106]^.

## THERAPEUTIC APPLICATIONS OF MOF NANOSYSTEM TO OVERCOME TUMOR RESISTANCE

### Improving delivery

**Figure 5:**
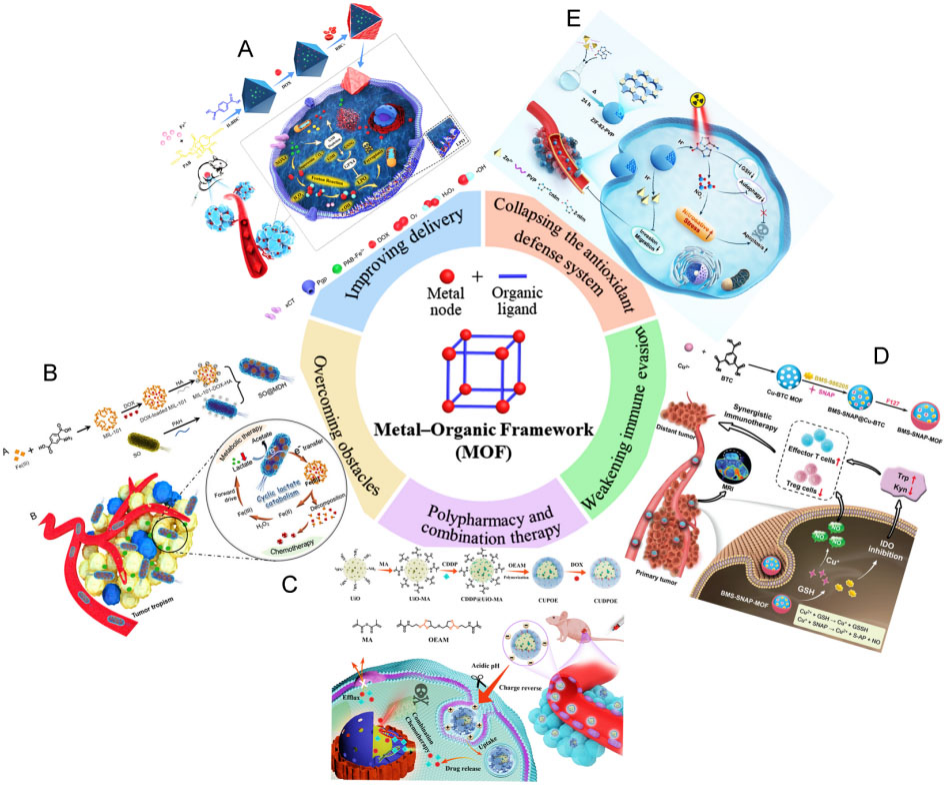
Summary of the therapeutic application of MOFs to overcome tumor resistance. (A) Improving delivery^[55]^. Reproduced with permission from ref^[55]^. Copyright 2021 the authors. (B) Overcoming obstacles^[40]^. Reproduced with permission from ref^[40]^ . Copyright 2021 American Chemical Society. (C) Polypharmacy and combination therapy^[36]^. Reproduced with permission from ref^[36]^. Copyright 2021 Elsevier. (D) Weakening immune evasion^[44]^. Reproduced with permission from ref^[44]^. Copyright 2022 Wiley-VCH. (E) Collapsing the antioxidant defense system^[118]^. Reproduced with permission from ref^[118]^. Copyright 2021 Wiley-VCH.

Normally, some drugs are consumed in the blood circulation by autoimmune system before they come to the tumor. The barriers from TME and cancer cells could work together to prevent drugs from entering the cells and even excrete drugs that have entered cells due to the overexpression of the drug efflux pump, making it difficult for the drugs to accumulate effectively in the cell. MOF-based NPs are allowed to accumulate in the tumor through enhanced permeability and retention effects (EPR)^[107]^. For example, Chen *et al.* developed an HA-coated Zr(IV)-based porphyrinic MOF DDS loaded with α-cyano-4-hydroxycinnamate (CHC) ^[35]^. This DDS was delivered to cancer cells with CD44 overexpression owing to the targeting ability of HA towards CD44, and then released Zr (IV)-based porphyrinic MOF and CHC for enhanced photodynamic therapy (PDT) effect. Du *et al.* synthesized DOX@NH_2_-MIL-88B-COD@CS NPs, which were loaded with cholesterol oxidase (COD), DOX, and chondroitin sulfate (CS) gel shell^[39]^. The COD catalyzed the degradation of cholesterol on the cell membrane to weaken the bio-barrier of cell membrane, thus facilitating the delivery of NPs into the cell. Meanwhile, H_2_O_2 _generated from the cholesterol degradation was catalyzed by the nanoenzyme, NH_2_-MIL-88B (MIL, Materials of Institute Lavoisier), to produce •OH to kill cancer cells. An erythrocyte membrane camouflaged iron-based MOF was developed for the multidrug delivery platform [Figure 5A]^[55]^. Erythrocyte membrane prolonged the circulation cycle of the multidrug platform in the blood as well as provided targeting. After endocytosis and catabolism, the multidrug platform released iron and multiple drugs, triggering the ferroptosis process and chemotherapeutic effect towards tumors. In addition, Cheng *et al.* utilized cell membrane artefacts from human breast cancer MDA-MB-231 and MOF to protect gelonin from catabolism^[108]^. The gelonin@ZIF-8/MDA-MB-231 (ZIF, zeolitic imidazolate framework) cell membrane was 11-fold more effective than free gelatin in cancer treatment. The biomimetic cascade MOF nanoreactor loaded with glucose oxidase (GOD) and DOX for starvation-amplified chemotherapy combination therapy demonstrated powerful anticancer efficacy^[109]^.

The efflux of intracellular drugs has been a difficult obstacle in the fight against MDR due to the overexpression of ATP-binding cassette transporter family proteins P-gp on cancer cell membranes. MOF-based NPs could enter the cell by endocytosis and ensure their release in the perinuclear region, bypassing membrane transporters. Innovating upon this evidence, Wang *et al.* showed a biohybrid bioreactor through the integration of DOX-loaded MIL-101 and a *Shewanella oneidensis* (SO) bacterium^[40]^. After injection, the bioreactor migrated to the tumor site with high lactic acid content. The metabolism of lactic acid in TME led to the degradation of MIL-101, the release of DOX, and the downregulation of P-gp, which was related to the tumor multidrug resistance, improving the therapeutic efficiency.

### Overcoming obstacles

The specific TME is resistant to the delivery and action of drugs^[48,110,111]^. PDAC has the highest mortality rate among various types of cancers owing to the high density of matrix which prevents drug penetration into the cancer cells^[48]^. Not only hypoxic environments but antioxidant defense systems mediate the resistance of PDT^[110]^. Some signaling, such as S100A9-CXCL12, creates a TME, rendering cancers insensitive to immunotherapy^[111]^. Even under high metabolic intensity, cancer cells tend to adopt the metabolism of glycolysis that consumed large amounts of oxygen and produced large amounts of lactate, resulting in a hypoxic state. The metabolite lactate promotes the development of drug resistance [Figure 5B]^[40]^. The hypoxic also greatly reduces the efficacy of kinetic therapies which rely on the conversion of ROS to stimulate oxidative stress. Intracellular autophagy and high levels of GSH lead to rapid degradation of drugs or resist the process of drug-induced cell death. Researchers have reversed resistance to enhanced therapy by increasing oxygen or ROS levels and depleting GSH. Since the drugs are susceptible to degradation, their activities could be maintained for a long time by the protection of a biocompatible and chemically stable MOF. We summarize the recent MOF-based materials to overcome cancer drug resistance in Table 1. For example, Lian *et al. *used PCN-333(Al) (PCN, porous coordination network) as the nanocarrier to load tyrosinase (TYR) for synthesizing an enzyme-MOF nanoreactor, converting the non-toxic prodrug paracetamol (APAP) to 4-acetamido-o-benzoquinone (AOBQ) *in vivo*^[33]^. They demonstrated that AOBQ induced the depletion of GSH and oxidative stress, attracting ROS-generated CDT to poison cancer cells. The cytotoxicity of enzyme from the enzyme-MOF nanoreactor was still observed after three days, while the free enzyme without MOF protection was completely inactive within a few hours. Pan *et al.* proposed a copper(II) nano-MOF loaded with disulfiram prodrug (DQ) and conjugated with GOD, displaying cascade oxidation and Fenton-like reaction^[7]^. The nano-MOF was first metabolized by GOD-catalyzed glucose to produce H_2_O_2_, which triggered the activation of the prodrug DQ to produce highly cytotoxic copper (II) diethyldithiocarbamate (Cu(DTC)_2_) *in situ* and Fenton-like reaction to generate •OH against cancer.

**Table 1:** A summary of MOFs to overcome cancer drug resistance

**MOFs**	**Drugs**	**Cancer treatment technologies**	**Methods of overcoming resistance**	**Reference**
Cu/ZIF-8	DNAzym, Cu^2+^	Gene therapy, chemotherapy	Drug intracellular activation, precise target	Li *et al.*^[43]^
CuTPyP	DOX, porphyrin	Chemotherapy, PDT	Efficient intracellular drug accumulation, cellular resensitization	Jiang *et al.*^[4]^
MIL (Fe)	DOX, Fe	Chemotherapy, CDT	Intracellular drug efflux inhibition, GSH consumption	Peng *et al.*^[55]^
MIL-101	DOX	Chemotherapy	Iintracellular drug efflux inhibition	Wang *et al.*^[40]^
MnMOFs	Cisplatin, Mn	Chemotherapy, microwave thermotherapy	GSH consumption, increasing of ROS levels	Wu *et al.*^[37]^
MOF-199	GOD, DQ	Chemotherapy, CDT	•OH generation, amplified effect	Pan *et al.*^[7]^
MOF-199	IDO, NO	Immunotherapy	Increasing of T cell infiltration	Du *et al.*^[44]^
NH2-MIL-88B	DOX, COD	Chemotherapy, CDT	Enhanced sensitivity of tumor cells to drug	Du *et al.*^[39]^
PCN-333 (Al)	TYR, AOBQ	Chemotherapy, CDT	*In vivo* enzymatic synthesis of drugs, precise target	Lian *et al.*^[33]^
Se/Ru-decorated porous MIL-101	siRNA	Gene therapy	Promoted siRNA escape from endosomes/lysosome	Chen *et al.*^[62]^
UiO-66-NH_2_	Cisplatin, DOX	Combination chemotherapy	Prolonged circulation process, efficient intratumoral accumulation	Hu *et al.*^[36]^
ZIF-8	Immunogenic dead cancer cells	Immunotherapy	Enhanced immunogenicity	Yang *et al.*^[45]^
ZIF-8	CQ, GOD	Starvation therapy	The autophagy inhibition	Li *et al.*^[47]^
ZIF-8	DOX, GOD	Starvation therapy, chemotherapy	TME adjustment, drug intracellular activation, precise target	Cheng *et al.*^[109]^
ZnMOF	DOX, quercetin	Chemotherapy	Efficient intracellular drug accumulation	Sun *et al.*^[38]^
Zr(IV)-based porphyrinic MOF	CHC	PDT	Reduced cellular O_2_ consumption, relieve hypoxia	Chen *et al.*^[35]^

DOX: doxorubicin; PDT: photodynamic therapy; CDT: chemodynamic therapy; GSH: glutathione; ROS: reactive oxygen species; AOBQ: 4-acetamido-o-benzoquinone.

### Polypharmacy and combination therapy

Single drugs and simple single-mechanism therapeutic effects are more likely to produce resistance in MDR. Researchers have found that unexpected efficacy can be achieved when a combination of multiple drugs acts simultaneously in the tumor^[112,113]^. Combination therapies with multi-drug synergistic effects or different mechanisms in tandem are becoming an effective way to improve tumor therapy. MOFs also show good applicability in constructing multidrug nanosystems. For example, Hu *et al.* synthesized hybrid NPs by employing porous UiO-66-NH_2_ (UiO, University of Oslo) as the nanocarrier and polymer as the shell to encapsulate cisplatin in MOF pores and DOX in the polymer shell, which showed high multidrug loading capacity and good biocompatibility [Figure 5C]^[36]^. The negatively charged shell was degraded in acidic TME and the positively charged MOF was then exposed to interact with the negatively charged cell membrane to promote drug intracellularization, lysosomal escape, and nuclear localization. This drug co-loaded hybrid nanosystem showed excellent anti-tumor effects and higher biosafety than the free drugs *in vivo*. In addition, Ling *et al.* synthesized a dual drug-loaded nanosystem in MOF with DOX and 5-fluorouracil, achieving good results in both anticancer therapy and bioimaging^[114]^. Jiang *et al. *synthesized a DOX-loaded MOF with porphyrin derivatives as organic ligands, combining chemotherapy with PDT^[4]^. DOX was shown to enhance the efficacy of PDT, causing more severe mitochondrial membrane potential damage and enhanced inhibition of P-gp.

### Weakening immune evasion

Immunotherapy is based on the patients immune systems^[115,116]^. By weakening the immune evasion of cancer cells, immunotherapy helps the immune system recognize tumors in the body and activate the immune program to clear cancer cells^[117]^. Immunotherapy has always been the most effective way to treat cancer with few side effects.

IDO plays a key role in mediating the immune evasion of cancer cells. The amino acid L-tryptophan (Trp) can be degraded to L-kynurenine (Kyn) catalyzed by IDO, which has a suppressive effect on cytotoxic T lymphocytes (CTL) as well as activates Treg cells to protect cancer cells. Du *et al.* synthesized a Cu-MOF to load with NO donor s-nitrosothiol groups (SNAP) and IDO inhibitor [Figure 5D]^[44]^. The abundant GSH triggered a cascade reaction to release drugs and produced NO *in situ*. These two substrates synergistically modulated the immunosuppressive TME accompanied with increasing CD8^+^ T cells, decreasing Treg cells, and weakening the immune evasion of the tumor. This immunotherapy showed significant anticancer effects. Safe, biocompatible, and physiologically stable MOFs show great potential as carriers of genetic material. For example, Chen *et al.* synthesized MIL-101(Fe) encapsulated with specific small interfering ribonucleic acids (siRNAs) and modified with selenium (Se)/ruthenium (Ru) NPs to protect siRNAs from nuclease degradation during targeted delivery into cancer cells^[62]^. The developed NPs promote cellular uptake and lysosomal escape of siRNA to silence the MDR gene. Previous kinetic therapies mainly killed cancer cells by elevating intracellular ROS to trigger oxidative stress and collapse the antioxidant defense system, as shown in Figure 5. The hypoxic environment is highly resistant to kinetic treatments such as PDT and CDT. However, hypoxia is ineffective against reactive nitrogen species (RNS) mechanisms as it does not require ROS involvement. Therefore, Li *et al.* utilized 2-nitroimidazole (2-nIm) and 1H-imidazole-4-cyano as ligands and Zn^2+^ as a metal node to synthesize a MOF. 2-nIm produced RNS to damage cancer cells under X-ray by exploiting the RNS mechanism, bypassing the resistance of the hypoxic environment [Figure 5E]^[118]^.

## FUTURE PERSPECTIVES

MOFs, formed by the coordination between metal ions or metal clusters and organic ligands, have become high-profile nanomaterials in medical research due to their controllable, regular structure, a large number of active sites, tumor microenvironment responsiveness, high biocompatibility, and physiological stability. Many researchers have devoted themselves to developing MOF-based DDS as nanocarriers of anticancer drugs. Although great progress has been made in related research, there are still many difficulties and challenges in the continuous development and translation of this emerging technology into clinical practice. First, the weak interactions between MOFs and drugs limit their development. Using MOFs as a nanocarrier to encapsulate or load anticancer drugs requires strong interactions between MOFs and anticancer drugs, such as the binding of special functional groups (carboxyl groups, aldehyde groups, etc.) and electrostatic adsorption caused by opposite charges. The weak interactions of MOF may result in drug leakage with reduced therapeutic efficiency. Second, the approaches to drug encapsulation into MOFs are limited. At present, there are three methods for the encapsulation of drugs and MOFs: one-pot synthesis, *in situ* encapsulation, and post-synthesis loading. *In situ* encapsulation is drug encapsulation during the synthesis of MOFs, which requires milder MOF synthesis conditions. However, not all MOF synthesis conditions allow *in situ* encapsulation, which limits the choice of carriers and drugs for DDS. Third, biocompatibility and safety issues of MOFs *in vivo* are uncertain. Although the biocompatibility of MOFs has been verified, a comprehensive evaluation of the *in vivo* toxicity of the DDS throughout the treatment process is still required before human therapy. Moreover, in fact, the function of MOFs as a nanocarrier is often to protect and release the drug in MOF-based DDS, while the functions such as targeting the tumor still need to rely on specific modifications.

In future studies, the development and practical application of MOF-based DDS need to overcome the above difficulties, design multifunctional MOFs, design mild synthesis conditions, construct novel MOFs with strong interrelationships between drugs, and evaluate the effects of DDS on organisms throughout the therapeutic process.

## CONCLUSION

The research progress of MOF-based anticancer DDS to overcome tumor drug resistance is summarized and reviewed. Among the tumor drug resistance features, biological barrier, drug inactivation, anti-apoptosis, DNA repair, and immune evasion, the tumor microenvironment with multiple resistance mechanisms cannot be ignored, which has a great impact on the drug resistance of tumors. Next, the synthesis and drug loading method of MOFs are described, which is crucial for the excellent drug loading and protection ability of MOFs. The advantages of MOFs and their application potential in drug delivery, bioimaging, and biomimetic catalysis are also demonstrated. Finally, the application of MOF-based DDS in chemotherapy, radiotherapy, immunotherapy, gene therapy, and starvation therapy, as well as combination therapy with various mechanisms, is systematically reviewed from multiple perspectives. The approaches of MOFs to protect drugs, overcome drug resistance, and improve the effectiveness of cancer treatment are expounded from four perspectives: improving drug entry into cancer cells, overcoming drug resistance barriers, multidrug loading and combination therapy, and reactivating immunotherapy. Compared with other nanocarriers, the tunable structure of MOFs provides great convenience for loading different drugs. The responsiveness of the tumor microenvironment provides a guarantee for drug protection and controlled release, which improves the efficiency of drug utilization. Using MOFs as nanocarriers to load drugs and overcome tumor resistance will become an emerging and achievable technology.

Benefiting from the controllable structure and high porosity of MOFs, based on the known drug resistance mechanisms, we can utilize biological and chemical techniques to load drugs on MOFs, and then modify them to synthesize composites for suppressing drug resistance of cancer cells and improving the efficacy of cancer therapy. Although there is still a certain gap from the actual human treatment and more intensive research is needed, we believe that MOFs will eventually overcome tumor drug resistance and show their great potential to benefit human cancer therapy.

## DECLARATIONS

### Copyright

© The Author(s) 2022.
